# Chlorogenic Acid Alleviates LPS-Induced Inflammation and Oxidative Stress by Modulating CD36/AMPK/PGC-1α in RAW264.7 Macrophages

**DOI:** 10.3390/ijms241713516

**Published:** 2023-08-31

**Authors:** Tiantian Gu, Zhiguo Zhang, Jinyu Liu, Li Chen, Yong Tian, Wenwu Xu, Tao Zeng, Weicheng Wu, Lizhi Lu

**Affiliations:** 1 State Key Laboratory for Managing Biotic and Chemical Threats to the Quality and Safety of Agro-Products, Institute of Animal Husbandry and Veterinary Science, Zhejiang Academy of Agricultural Science, Hangzhou 310021, China; gutiantian1029@outlook.com (T.G.); ljy52559@outlook.com (J.L.); cl0010@outlook.com (L.C.); tyong@zaas.ac.cn (Y.T.); xuwenwu248@outlook.com (W.X.); zengtao@zaas.ac.cn (T.Z.); 2 Food Science Institute, Zhejiang Academy of Agricultural Sciences, Hangzhou 310021, China; zhangkii@126.com

**Keywords:** chlorogenic acid, oxidative stress, inflammation, CD36/AMPK/PGC-1α

## Abstract

Chlorogenic acid (CGA) is a bioactive substance with anti-inflammatory activities. Clusters of CD36 have been suggested to be widely involved in inflammatory damage. However, the mechanism of CGA protecting against LPS-induced inflammation involving the CD36 regulation is unclear. Here, we demonstrated that CGA protected against LPS-induced cell death and decreased the production of ROS. Moreover, the SOD, CAT, and GSH-Px activities were also upregulated in CGA-treated cells during LPS stimulation. CGA reduced COX-2 and iNOS expression and IL-1β, IL-6, and TNF-α secretion in LPS-stimulated RAW264.7 macrophages. In addition, CGA treatment widely involved in immune-related signaling pathways, including NF-κB signaling, NOD-like receptor signaling, and IL-17 signaling using transcriptomic analysis and CD36 also markedly reduced during CGA pretreatment in LPS-induced RAW264.7 cells. Furthermore, the CD36 inhibitor SSO attenuated inflammation and oxidative stress by enabling activation of the AMPK/PGC-1α cascade. These results indicate that CGA might provide benefits for the regulation of inflammatory diseases by modulating CD36/AMPK/PGC-1α to alleviate oxidative stress.

## 1. Introduction

Chlorogenic acid (CGA) is a bioactive substance that widely exists in natural food [1]. CGA has been shown to have pharmacological effects due to its immune-regulating, antioxidant, anti-inflammatory, anti-carcinogenic, and anti-bacterial activities [2]. Studies suggest that CGA is especially effective for the prevention of diseases resulting from inflammation [3]. The mechanism underlying these effects is unclear.

Inflammation is a crucial physiological and immune response of tissues and cells against pathogenic challenges [4]. The movement and gathering of neutrophils during inflammation is significant because proinflammatory cytokines released by macrophages cause endothelial cells to express adhesion proteins and promote leukocyte motility [5]. Macrophages are the main pro-inflammatory cells that can protect the body from external intruders by synthesizing inflammatory cytokines such as tumor necrosis factor-alpha (TNF-α), interleukin (IL)-6 and IL-1β [6,7,8]. Improper activation or abnormal upregulation of the cyclooxygenase-2 (COX-2) is often seen in inflammatory diseases [9].

Reactive oxygen species (ROS) function as signaling molecules in host defense responses. Oxidative stress can result from an excessive buildup of ROS in inflammatory processes [10,11]. Expression of inducible nitric oxide synthase (iNOS) promotes ROS generation and is often positively correlated with proinflammatory cytokine expression [12,13]. Antioxidants decrease ROS by scavenging ROS to activate the endogenous enzymatic antioxidant system in cells and organ systems [14,15,16]. For instance, the antioxidant enzymes superoxide dismutase (SOD) and catalase (CAT) scavenge ROS to regulate intracellular ROS levels and protect cells from oxidative damage [17]. Kuo et al. showed that oxidative stress leads to the overproduction of ROS and decreased expression of antioxidative enzymes that protect tissues in vivo [15].

Cluster of differentiation 36 (CD36) is a scavenger receptor that is densely expressed on the surface of macrophages and has well-known roles in immunity, metabolism, and atherogenesis [18]. There is increasing evidence that CD36 participates in the inflammatory response and mitochondrial fatty acid oxidation, which contributes to the regulation of chronic metabolic diseases [19,20]. In this process, CD36 mediates inactivation of AMP-activated protein kinase (AMPK) signaling to regulate oxidative metabolism [21]. Key to cellular metabolism’s energy sensing, AMPK also plays a role in inflammation, oxidative stress, neurodegeneration, and other types of metabolic stress [22]. In a mouse model induced by LPS, emerging data suggests that AMPK performs protective roles as a negative regulator of inflammatory reactions [23].

In this study, we aimed to investigate the anti-inflammatory action of CGA and its underlying molecular mechanism in LPS-induced RAW264.7 macrophages. We hypothesized that CGA would attenuate LPS-induced inflammation and oxidative stress by modulating the CD36/AMPK/PGC-1α. Our results demonstrate that CGA negatively regulates CD36, resulting in activation of an AMPK-dependent anti-inflammatory pathway. This suggests that CGA has the potential to ameliorate inflammatory effects via the CD36/AMPK/PGC-1α in inflammation-related diseases.

## 2. Results

### 2.1. CGA Alleviated the Cell Viability Induced by LPS

**Figure 1:**
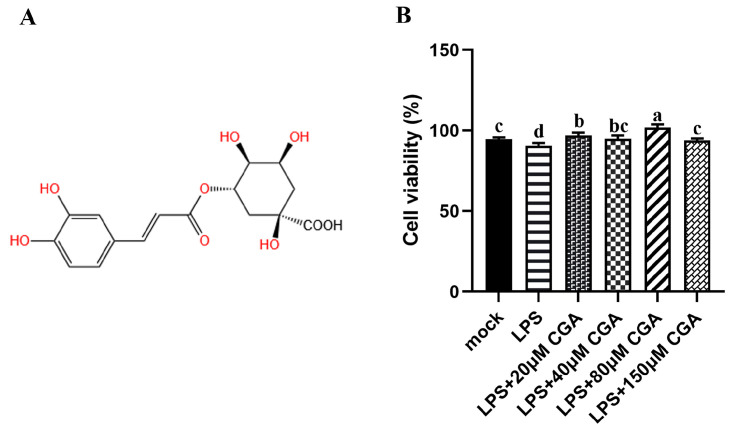
Effect of chlorogenic acid (CGA) on cell proliferation. (**A**) The chemical structure of CGA. (**B**) CCK-8 assays of cell proliferation. Results are shown as the mean ± SD (*n* = 6). Different letters indicate significant differences between groups (*p* < 0.05).

The structure of chlorogenic acid is presented in Figure 1A. The toxicity of CGA in RAW264.7 macrophages was evaluated by CCK-8 assays (Figure 1B). The difference in viability between LPS-induced and mocked RAW264.7 cells showed significance in that the proliferation of cells was markedly reduced under LPS induction. CGA treatment enhanced the proliferation of cells in the groups treated with LPS, especially at a concentration of 80 μM (Figure 1B). These results suggested that CGA mitigated LPS-induced cell death.

### 2.2. CGA Reduced LPS-Induced Oxidative Stress

**Figure 2:**
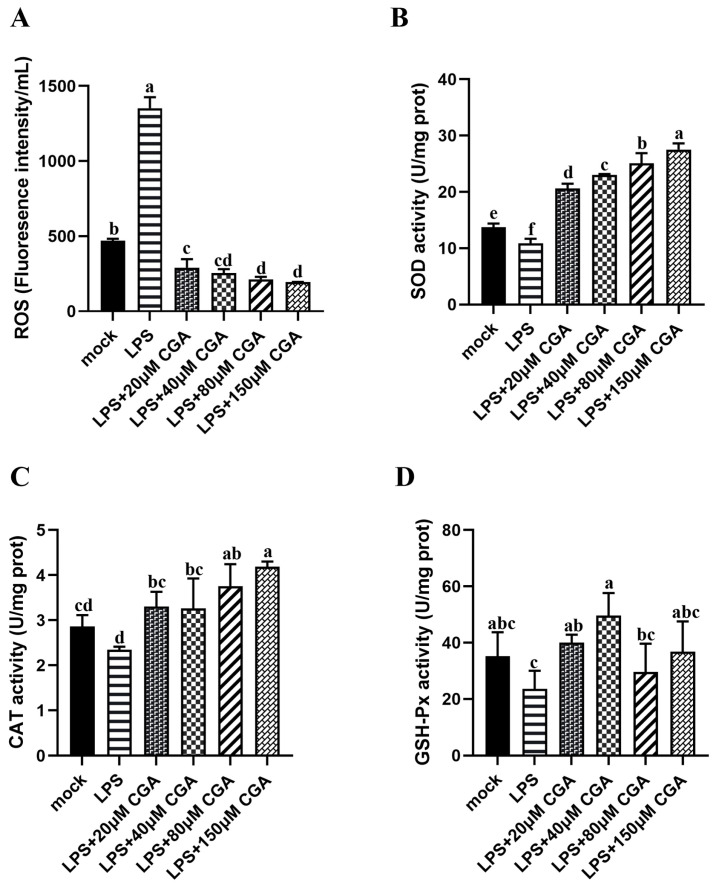
CGA reduced LPS-induced oxidative stress. (**A**) ROS production. (**B**) The activity of SOD. (**C**) The activity of CAT activity, and (**D**) the activity of GSH-Px activity in different groups. Results are shown as the mean ± SD (*n* = 3). Different letters indicate significant differences between groups (*p* < 0.05).

To explore the effects of CGA on the anti-oxidative stress response, ROS, SOD, CAT, and GSH-Px levels were determined. The concentrations of 20–150 μM CGA could significantly inhibit the generation of ROS in LPS-induced cells (Figure 2A). LPS induced a significant decrease in SOD, CAT, and GSH-Px activities, while CGA treatment significantly mitigated cell injury induced by LPS, which showed higher significance in these three anti-oxidative substances (Figure 2B–D). In general, CGA alleviated the oxidative stress induced by LPS by promoting the production of antioxidizing substances.

### 2.3. CGA Reduced LPS-Induced Inflammatory Mediators and Cytokines Expression

**Figure 3:**
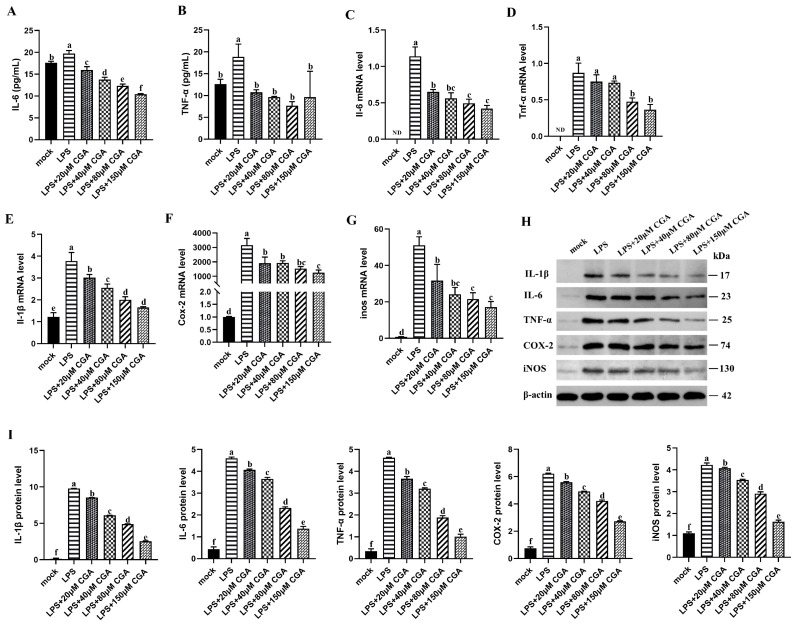
CGA reduced LPS-induced inflammatory mediators and cytokines expression. (**A**,**B**) The secretion of IL-6 and TNF-α. (**C**–**G**) mRNA level and (**H**,**I**) protein expression of IL-6, TNF-α, IL-1β, COX-2, and iNOS. Results are shown as the mean ± SD (*n* = 3). Different letters indicate significant differences between groups (*p* < 0.05).

As shown in Figure 3A, treatment with LPS markedly induced IL-1β production compared with that in mock-treated cells; however, pretreatment with 40, 80, or 150 μM CGA effectively suppressed the LPS-induced IL-6 and TNF-α levels in LPS-stimulated cells (Figure 3A,B). To further confirm the regulation of CGA in the anti-inflammatory response, we determined the protein and mRNA expressions of inflammatory factors. Compared with mocked cells, the cells treated with LPS had significantly increased mRNA levels of *Il-6*, *Tnf-α*, and *Il-1β* (Figure 3C–E). Similarly, LPS treatment resulted in increased mRNA levels of *COX-2* and *iNOS*, which was significantly attenuated by CGA (Figure 3F,G). The results were similar at the protein level; CGA suppressed LPS-induced protein levels of IL-1β, IL-6, TNF-α, COX-2, and iNOS in RAW264.7 macrophages (Figure 3H,I).

### 2.4. Transcriptomic Analysis Reveals That CGA Alleviates LPS-Induced Inflammation and Oxidative Stress by Gene Regulation

**Figure 4:**
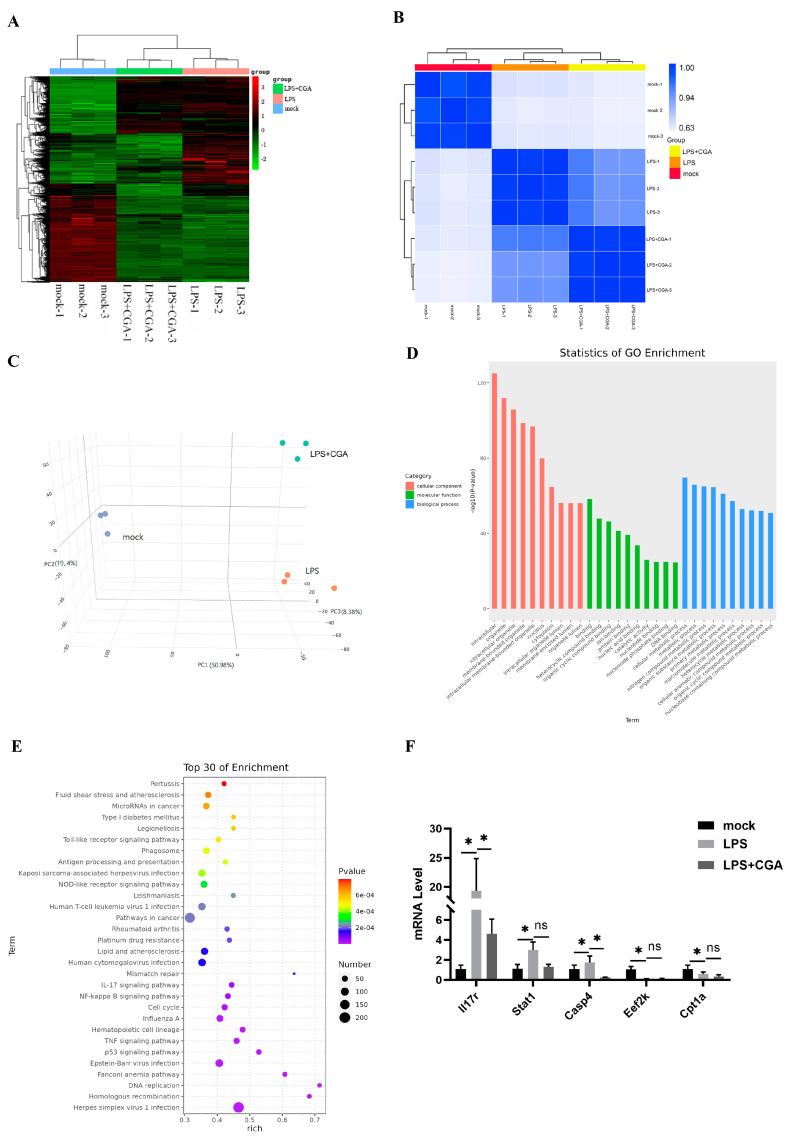
Transcriptomic analysis reveals that CGA alleviates LPS-induced inflammation and oxidative stress by gene regulation. (**A**) A heatmap of differentially expressed genes. (**B**) Correlation analysis. (**C**) Principal component analysis of the samples of mock−treated, LPS−treated, and CGA+LPS−treated cells. (**D**) Gene ontology analysis results. (**E**) KEGG analysis results. (**F**) qPCR validation of selected genes. Different letters (a, b, c) indicate significant differences between groups (*p* < 0.05). Results are shown as the mean ± SD (*n* = 3). Different letters and * indicate significant differences between groups (*p* < 0.05).

To evaluate the comprehensive mechanism of CGA on anti-inflammation and anti-oxidative stress, we systematically compared the transcriptomes of mock−treated cells, LPS−treated cells, and cells co-treated with LPS and CGA (80 μM CGA for 4 h and 1 μg/mL LPS for 24 h). We identified over 5800 DEGs among the three treatment groups, including 4630 DEGs between the mock-treated cells and LPS−treated cells (2513 upregulated and 2117 downregulated in the LPS-treated cells) and 1189 DEGs between the LPS−treated cells and LPS+CGA−treated cells (72 upregulated and 1117 downregulated in the LPS+CGA−treated cells; Figure S1). These results were confirmed by heatmap and correlation analysis, which exhibited the gradual change in the characteristics of CGA in LPS−induced cells (Figure 4A,B). PCA analysis showed that all the samples were well clustered within their own groups (Figure 4C). GO analysis revealed that the top enriched terms were mostly related to extracellular regulation, including extracellular organelles and extracellular membrane-bound organelles (Figure 4D). KEGG analysis revealed that many pathways are involved in immunoregulation, including NF-κB signaling, TNF signaling, p53 signaling, NOD-like receptor signaling, and IL-17 signaling (Figure 4E). RT-qPCR was used to detect the immunoinflammatory marker genes *Il7r* and *Stat1* and the apoptosis-related gene *Casp4* showed that CGA was effective in relieving inflammation (Figure 4F).

### 2.5. CGA Treatment Reduced CD36 Expression

**Figure 5:**
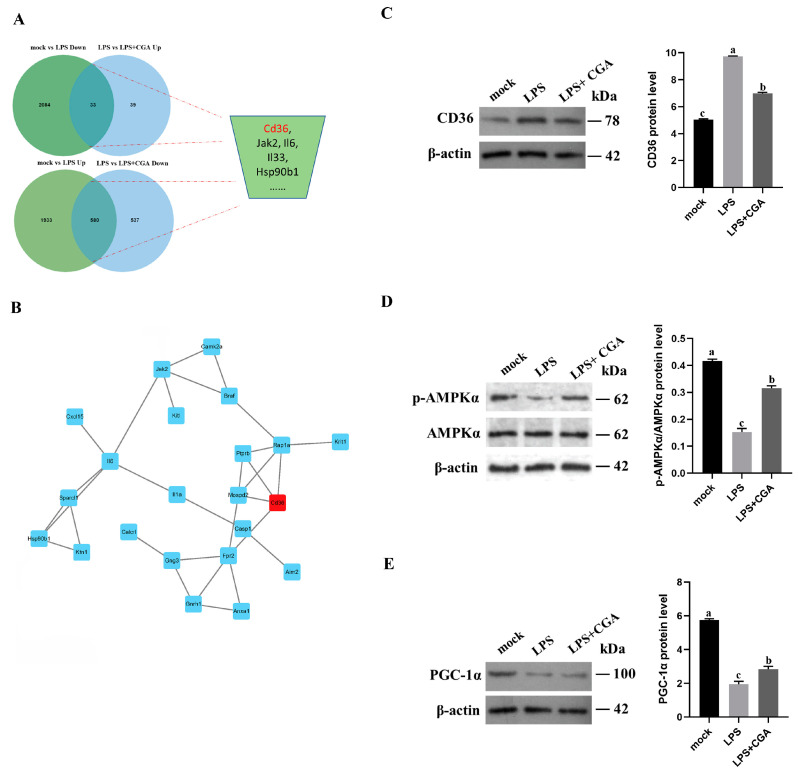
CGA treatment inhibited CD36 expression. (**A**) A Venn analysis of mock-treated vs. LPS−treated cells and LPS−treated vs. LPS+CGA−treated cells with 613 shared DEGs with opposite trends selected. (**B**) Protein–protein interaction analysis of CD36. Red indicated *Cd36*. (**C**–**E**) The protein expression of CD36, phosphorylated AMPK, and PGC-1α. Results are shown as the mean ± SD (*n* = 3). Different letters indicate significant differences between groups (*p* < 0.05).

We performed a Venn analysis to identify DEGs whose expression was changed in opposite directions by LPS treatment and CGA treatment. A total of 613 DEGs showed opposite trends in comparisons between mocked cells vs. LPS−treated cells and LPS−treated cells vs. LPS+CGA−treated cells (Figure 5A and Figure S1). A protein–protein interaction analysis of *Cd36* in the selected DEGs identified interactions of *Cd36* with *Mospd2*, *Rap1a*, *Ptprb*, and *Fpr2* (Figure 5B). Subsequently, we evaluated CD36 protein expression in LPS−treated cells, which showed that CD36 expression was higher during LPS stimulation than in untreated cells and CGA effectively inhibited the CD36 expression in LPS−induced cells (Figure 5C). In addition, CGA increased the expression of phosphorylated AMPK and PGC-1α in LPS−induced cells (Figure 5D,E).

### 2.6. CD36 Inhibition Enabled Anti-Inflammation and Oxidative Stress through the CD36/AMPK/PGC-1α Cascade

**Figure 6:**
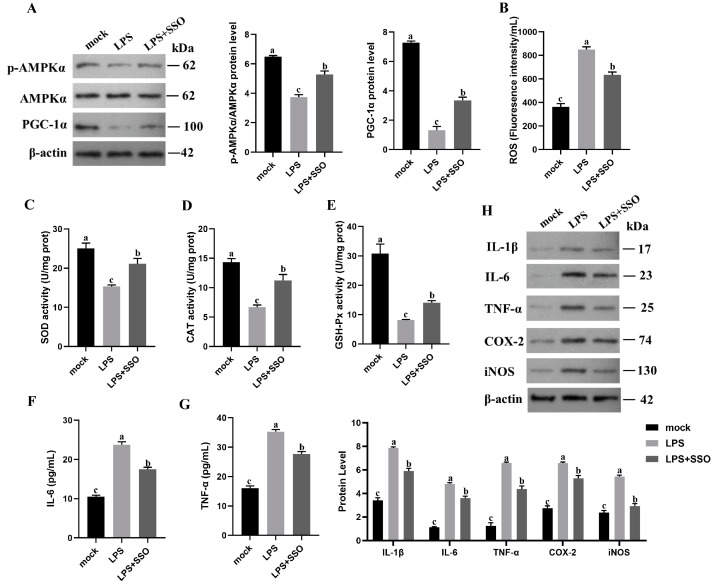
CD36 inhibition defended anti-inflammatory and oxidative stress functions through the CD36/AMPK/PGC-1α cascade. (**A**) The phosphorylated AMPKα and PGC-1α protein expression. (**B**) ROS levels. (**C**–**E**) Oxidative stress parameters (SOD, CAT, and GSH-Px activities). (**F**,**G**) Inflammatory cytokine (IL-6 and TNF-α) expression. (**H**) Inflammatory mediators and cytokines (IL-1β, IL-6, TNF-α, COX-2, and iNOS) protein expression. Results are shown as the mean ± SD (*n* = 3). Different letters indicate significant differences between groups (*p* < 0.05).

To determine whether CD36 can affect the AMPK signaling pathway to regulate LPS−induced inflammation, we treated LPS−induced RAW264.7 cells with SSO, an inhibitor of CD36. SSO increased the levels of PGC-1α and phosphorylated AMPKα and reduced ROS production in LPS−induced cells (Figure 6A). SSO treatment also inhibited the generation of ROS during LPS stimulation (Figure 6B). In addition, SSO pretreatment effectively attenuates the activities of SOD, CAT, and GSH-Px during LPS stimulation (Figure 6C–E). Furthermore, LPS−induced increases in inflammatory cytokine levels were also attenuated by pretreatment with SSO (Figure 6F,G). Western blots confirmed that SSO pretreatment attenuated LPS-induced increases in COX-2 and iNOS protein expression and IL-1β, IL-6, and TNF-α production (Figure 6H). These results suggest that CD36 suppression effectively alleviates LPS−induced oxidative stress and inflammation by modulating the CD36/AMPK/PGC-1α cascade.

## 3. Discussion

Numerous diseases and health issues are strongly related to inflammation, which the body uses as a defense mechanism. Many studies have focused on finding safe candidate materials to prevent and treat inflammatory diseases through diverse inhibitory actions. CGA has potential anti-inflammatory activities that may prevent the progression of inflammatory-related diseases [24]. The present study aimed to evaluate the underlying mechanism of CGA in anti-inflammatory effects. CGA treatment did not inhibit cell viability, which suggests that it may have a few unwanted side effects. Importantly, CGA exhibited strong anti-inflammatory activity in LPS-induced RAW264.7 macrophage cells by downregulating the secretion of inflammatory cytokines and mediators such as IL-1β, IL-6, TNF-α, COX-2, and iNOS, which is consistent with previous findings [25].

Studies have reported that excessive ROS accumulation could decrease antioxidant defenses and lead to oxidative stress, which plays an important role in the inflammatory process [26,27]. ROS are required for critical physiological processes and behaviors. However, they serve as inflammatory mediators in the development of inflammatory illnesses, and excessive generation of ROS and related species is one of the causes of oxidative stress [28]. We found that treatment with CGA considerably reduced the production of ROS in LPS−induced cells. We further confirmed that CGA reduced SOD, CAT, and GSH-Px levels in the LPS−induced cells, suggesting that CGA plays an important function in the anti-oxidative stress on LPS−induced inflammation.

We used high-throughput RNA-seq to determine possible molecular mechanisms by which CGA ameliorates LPS-induced oxidative stress in inflammatory processes. We identified >5800 DEGs in comparisons between mock−treated and LPS−treated cells and between LPS−treated and LPS+CGA−treated cells. KEGG analysis results suggested that these DEGs were mainly enriched in pathways for NF-κB signaling, TNF signaling, p53 signaling, NOD-like receptor signaling, and IL-17 signaling. Among the identified DEGs, we selected the genes that showed opposite trends in response to LPS treatment and CGA treatment. CD36 stood out among the selected genes and aroused our interest because of its role in immunity [24].

As one member of the class B scavenger receptor family, CD36 is distributed in various types of cells and mediates immunological recognition, inflammation, molecular adhesion, and apoptosis [29]. Overexpression of CD36 is correlated with chronic inflammation, tumorigenesis, and metabolic dysfunction [30,31]. Disruption of CD36 in oleic acid-induced HeLa cells as well as mice models significantly inhibited cervical cancer carcinogenesis by downregulating the Src/ERK pathway in vitro and in vivo [32]. Moreover, Sun et al. reported that the loss of CD36 restored LPS−induced acute lung injury attenuated by attenuating NF-κB activation in macrophages [33]. We found that the mRNA and protein expression of CD36 was significantly increased in LPS−induced cells compared with mocked cells, indicating that inflammatory processes were enhanced in the LPS−induced cells. CGA treatment reduced the LPS−induced expression of CD36 and thereby inhibited inflammation, indicating that anti-inflammatory processes were enhanced by CGA. In a previous study, the absence of CD36 as a key regulatory molecule attenuated ROS production in response to inflammatory diseases [34]. We confirmed that pretreatment with SSO, an inhibitor of CD36, reduced ROS production and inhibited the expression of IL-1β, IL-6, TNF-α, COX-2, and iNOS in LPS−induced RAW264.7 cells. These results indicated that CD36 effectively participates in LPS−induced oxidative stress and inflammatory effects.

**Figure 7:**
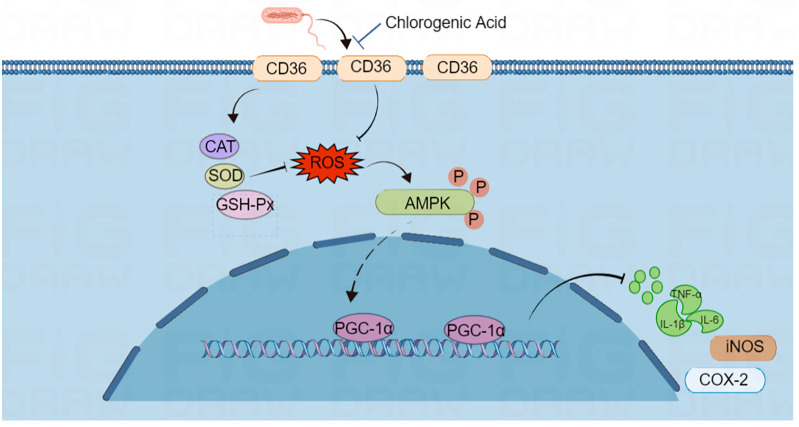
CGA reduces LPS−induced oxidative stress and inflammation by activating the CD36/AMPK/PGC-1α pathway.

AMPK is a highly conserved serine/threonine protein kinase that can be regulated by ROS levels in mitochondria [35]. AMPK acts together with its downstream targets to upregulate PGC-1α [19] and thereby help control mitochondrial biosynthesis, energy metabolism, and inflammation as a homeostasis-sensing network. We found that LPS−induced ROS production suppresses AMPK phosphorylation, leading to the deactivation of AMPK, whereas CGA treatment protects AMPKα phosphorylation in LPS−induced macrophages. In a previous study, AMPK activation was highly related to the effect of inflammation [36]. We, therefore, considered AMPK as a potential mediator that could regulate the inflammatory status. CGA upregulates PGC-1α by inhibiting CD36, thus decreasing the expression levels of IL-1β, IL-6, TNF-α, COX-2, and iNOS to elicit biological effects. These data suggest that CGA prevents inflammatory responses, and this effect might be mediated by modulation of the CD36/AMPK/PGC-1α cascade (Figure 7).

## 4. Materials and Methods

### 4.1. Regents and Materials

Chlorogenic acid extracted from sweet potato leaf was obtained from the Food Science Institute, Zhejiang Academy of Agricultural Sciences. Commercial assay kits for IL-1β, TNF-α, and IL-6 were purchased from R&D Systems (Minneapolis, MN, USA). Cell-Counting Kit-8 (CCK-8) was purchased from Vazyme (Wuhan, China). FastQuant RT Kit with gDNA and TB Green^®^ Premix Ex Taq™ II were obtained from Tiangen (Beijing, China) and TaKaRa (Dalian, China), respectively. BCA Protein Assay Kit was obtained from Beyotime (Shanghai, China). Mouse polyclonal antibodies to IL-1β, IL-6, TNF-α, COX-2, CD36, PGC-1α, and β-actin were obtained from Abcam (Cambridge, UK). Mouse polyclonal antibodies to iNOS, AMPKα, and p-AMPKα were obtained from CST (Boston, MA, USA). N-succinimidyl oleate (SSO) was purchased from MCE (Shanghai, China).

### 4.2. Cell Culture

RAW264.7 cells were purchased from Procell Life Science & Technology Co., Ltd. (Wuhan, China). Cells were cultured in DMEM (Gibico, Carlsbad, CA, USA) in supplementation with 10% fetal bovine serum (Gibico, Carlsbad, CA, USA) at 37 °C with 5% CO_2_ under sterile conditions.

### 4.3. Cell Viability Assay

The proliferation of RAW264.7 cells was measured by Cell Counting Kit-8 (CCK8, Vazyme, Nanjing, China). In brief, cells were plated on 24-well plates overnight. When incubated, 50 μL CCK-8 solution was added to the cell suspension, and the cells were incubated for another 4 h for color development. Finally, the proliferation of cells was detected by measuring absorbance using a multifunctional microplate reader (Tecan Infinite M200 PRO; Männedorf, Switzerland) at 450 nm.

### 4.4. Reactive Oxygen Species Detection

After treatment, the cells were collected, and the ROS level (fluorescence intensity/mL) was determined by undergoing 2,7-dichlorofluorescein diacetate (DCFH-DA) assay. In brief, the cell suspension was mixed with 10 μM diluted regent at 37 °C for 30 min. The cells were then detected by fluorescence microplate reader with an excitation wavelength of 488 nm and an emission wavelength of 525 nm; all samples were detected with multiple readings (Tecan, Mannedorf, Switzerland).

### 4.5. Determination of GSH-Px, SOD and CAT Activities

The cells were collected and treated by ultrasonic wave, and then the cells were centrifuged at 8000× *g*, 4 °C for 10 min to collect the supernatant. Finally, the CAT (U/mg.prot) activities were detected with wavelength of 405 nm, and the GSH-Px (U/mg.prot) and SOD (U/mg.prot) activities were detected with wavelength of 340 nm using microplate reader (Tecan) following the manufacturer’s instructions.

### 4.6. Enzyme-Linked Immunosorbent Assay (ELISA)

RAW264.7 cells were treated with different concentrations of CGA or 100 μM SSO for 4 h and then stimulated with 1 μg/mL LPS for 24 h. The culture supernatants were collected after centrifugation at 1000 rpm for 5 min at 4 °C and subjected to double antibody sandwich ELISA for detection of TNF-α, and IL-6 with ELISA kits according to the manufacturer’s recommendations. The concentrations of TNF-α (pg/mL), and IL-6 (pg/mL) in the samples were detected at the absorbance of 490 nm with a multifunctional microplate reader (Tecan Infinite M200 PRO; Tecan, Männedorf, Switzerland).

### 4.7. Real-Time Quantitative PCR (RT-qPCR)

Total RNA was isolated from cell samples using TRIzol reagent (Invitrogen) and used to synthesize cDNA with a FastQuant RT Kit (With gDNase). RT-qPCR was conducted in a LightCycler^®^ 96 Real-Time PCR System with TB Green PCR Master Mix. Reactions were initiated by incubation at 95 °C for 30 s, 40 cycles of 95 °C for 5 s, and 60 °C for 20 s. The 18s gene was used as an internal reference gene and the relative expression level was calculated using the 2^−ΔΔCT^ method. The gene primers for RT-qPCR are provided in Table 1.

### 4.8. Western Blot

Cells were collected after incubation. Then, total protein was extracted using lysis buffer and quantified using BCA Protein Assay Kits. A 10% SDS-PAGE gel was used to separate the protein samples, and the gels were transferred to polyvinylidene fluoride (PVDF) membranes (Bio-Rad, California, USA). The membranes were blocked in 5% non-fat milk with Tween-20 buffer for 1 h and then incubated with primary antibodies overnight at 4 °C. Following 1 h incubation with secondary antibodies at room temperature, ECL detection was performed on the membranes.

### 4.9. Transcriptomics and Validation

Three biological replicates of mocked, LPS−treated, or LPS+CGA−treated RAW264.7 cells were collected. Total RNA extraction, library construction, sequencing, and differential expression analysis were conducted by Personal Biotech (Shanghai, China). Differentially expressed genes (DEGs) were screened as |Fold Change| ≥ 1.5 and FDR < 0.05. Gene Ontology (GO) and Kyoto Encyclopedia of Genes and Genomes (KEGG) pathway enrichment of DEGs was implemented by R software. A protein–protein interaction network was constructed using the stringApp(ver.1.7.1) in Cytoscape(ver. 3.9.1). Five significant DEGs were randomly chosen for validation by RT-qPCR using specific primers synthesized by Youkang Company (Hangzhou, China) (Table 1).

### 4.10. Statistical Analysis

Data were presented as the mean ± standard deviation (SD). All significance analyses were determined using Duncan in SPSS 25.0 (SPSS Inc.; Chicago, IL, USA). For all comparisons, *p* < 0.05 was considered significant difference. All comparisons were performed using GraphPad Prism 8.0 and the flow chart was drawn with Figdraw.

## 5. Conclusions

In summary, CGA alleviates the ROS level and upregulates CAT, SOD, and GSH-Px activities in LPS−treated RAW 264.7 macrophages. CGA played positive roles in LPS−induced inflammation in RAW 264.7 macrophages. Furthermore, CGA suppressed LPS−induced inflammation and oxidative stress by regulating CD36/AMPK/PGC-1α cascade *in vitro*, suggesting that CGA might provide benefits for the regulation of oxidative inflammatory diseases. These results represent that CGA has the potential to ameliorate inflammatory effects via the CD36/AMPK/PGC-1α in inflammation-related diseases.

## Figures and Tables

**Table 1:** Primer sequences used in RT-qPCR analysis.

Gene		Primer Sequences (5′ to 3′)	Annealing (°C)
*18s*	Forward (sense)	GTAACCCGTTGAACCCCATT	60
	Reverse (antisense)	CCATCCAATCGGTAGTAGCG	
*Il-1β*	Forward (sense)	GGCAGGCAGTATCACTCATTGTG	60
	Reverse (antisense)	GCTCATGTCCTCATCCTGGAAG	
*Il-6*	Forward (sense)	TCTACTCGGCAAACCTAGTGCGTTA	60
	Reverse (antisense)	TTCTGACCACAGTGAGGAATGTCCA	
*Tnf-α*	Forward (sense)	GACCCTCACACTCAGATCATCTTCT	60
	Reverse (antisense)	GCTACGACGTGGGCTACAG	
*COX-2*	Forward (sense)	GGCAGGAAGTCTTTGGTCTGGT	60
	Reverse (antisense)	CTGGTTTGGAATAGTTGCTCATCAC	
*iNOS*	Forward (sense)	TGCCACGGACGAGACGGATA	60
	Reverse (antisense)	AGGAAGGCAGCGGGCACAT	
*Il7r*	Forward (sense)	GCATCCCCAACCAACTGAGG	60
	Reverse (antisense)	GCATCTTCTAGGTCTCTTTTGAGC	
*Casp4*	Forward (sense)	TGTCTCATGGCACACTGCAT	60
	Reverse (antisense)	TTCTCCAGAGTTCCCACCTC	
*Cpt1a*	Forward (sense)	TCGGTGAGCCTGGCCT	60
	Reverse (antisense)	TTGAGTGGTGACCGAGTCTG	
*Eef2k*	Forward (sense)	CACCTGGAAGATATTGCCACC	60
	Reverse (antisense)	GCTTCGCCACGTAGTTGGA	
*Stat1*	Forward (sense)	CGCTGCCTATGATGTCTC	60
	Reverse (antisense)	TTTCCGTATGTTGTGCTG	

## Data Availability

The RNA sequencing read data were deposited in the GenBank SRA database under the accession number PRJNA962608.

## Supplementary Materials

The following supporting information can be downloaded at: https://www.mdpi.com/article/10.3390/ijms241713516/s1.
