# Supplementary figures and images for: Chlorogenic Acid Alleviates LPS-Induced Inflammation and Oxidative Stress by Modulating CD36/AMPK/PGC-1α in RAW264.7 Macrophages
Source: Int J Mol Sci. 2023 Aug 31;24(17):13516. doi: 10.3390/ijms241713516 (PMC10487601; doi:10.3390/ijms241713516)

**A**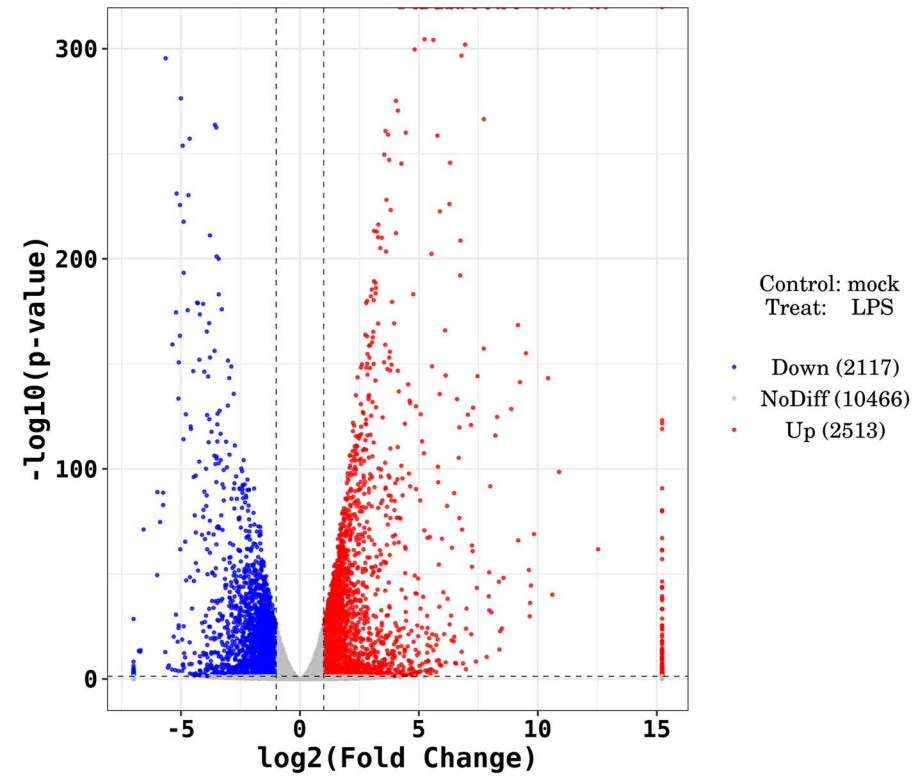**B**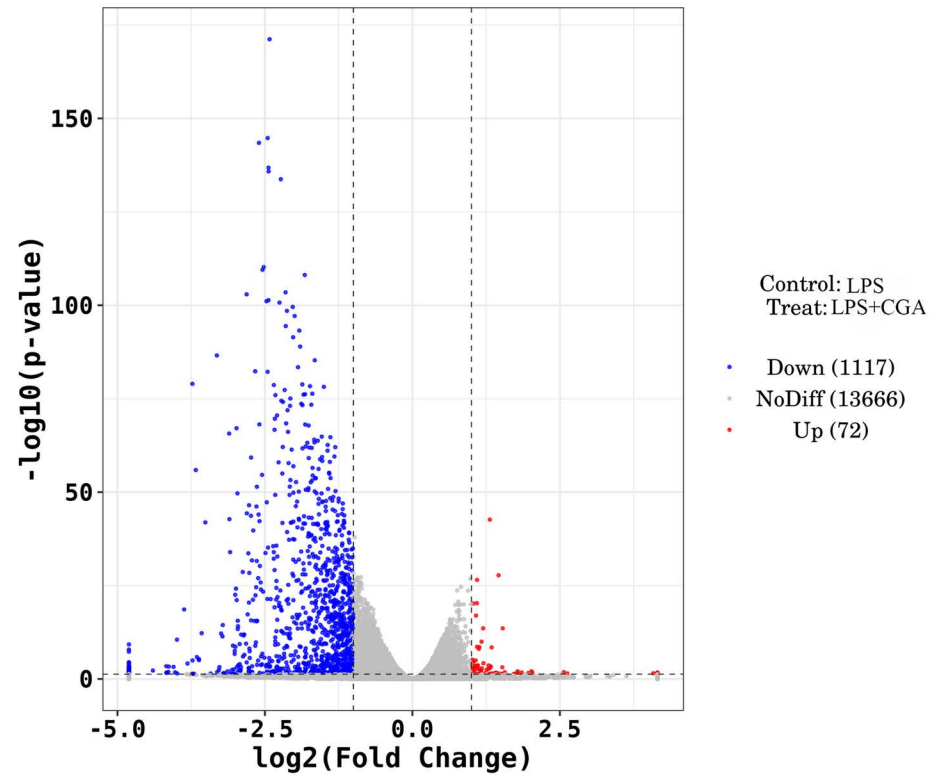

Supplement: Supplementary file 1 [file ijms-24-13516-s001.zip › ijms-2555710 supplementary.pdf]
